# Progressive gut microbiota shifts and functional alterations across aging stages and frailty in mice

**DOI:** 10.1016/j.isci.2025.112985

**Published:** 2025-06-23

**Authors:** Xinwei Jiao, Hongyu Li, Ting Wang, Hongchen Fu, Shiwu Wang, Hong Liu, Lei Wang, Xiuyun Li, Aijun Deng, Zhijie Li

**Affiliations:** 1 Department of Ophthalmology, Affiliated Hospital of Shandong Second Medical University, School of Clinical Medicine, Shandong Second Medical University, Weifang 261053, China; 2 Department of Ophthalmology, Zhengzhou University People’s Hospital, Henan Provincial People’s Hospital, Zhengzhou 450003, China

**Keywords:** Microbiology, Microbiome

## Abstract

The gut microbiota modulates aging through metabolic and immune interactions. To characterize microbial changes during aging, we profiled gut microbiota across four stages in male C57BL/6J mice: young-adult, middle-aged, senescent, and frailty. Using 16S rRNA sequencing and targeted metabolomics, we observed a biphasic diversity pattern, with reduced richness in senescence followed by a rebound in frailty. Frailty-stage microbiota showed increased connectivity and enrichment of *Lachnospiraceae*, alongside short-chain fatty acid (SCFA) depletion, particularly butanoic acid. Functional predictions revealed elevated immune activity and reduced carbohydrate metabolism, linked to specific microbial taxa. These findings highlight a structured remodeling of the gut microbiota during aging, with frailty representing a distinct dysbiotic state associated with metabolic dysfunction. Modulating gut microbial composition may represent a potential strategy to attenuate frailty and promote healthy aging.

## Highlights

• Gut microbiota diversity declines in senescence and rebounds in frailty
• *Lachnospiraceae* dominates frailty microbiota and drives increased network complexity
• Frailty shows reduced SCFAs and enriched immune and aging-related pathways
• Butanoic acid is depleted and linked to *Lachnospiraceae* and *Alistipes* abundance

## Graphical abstract

**Figure:**
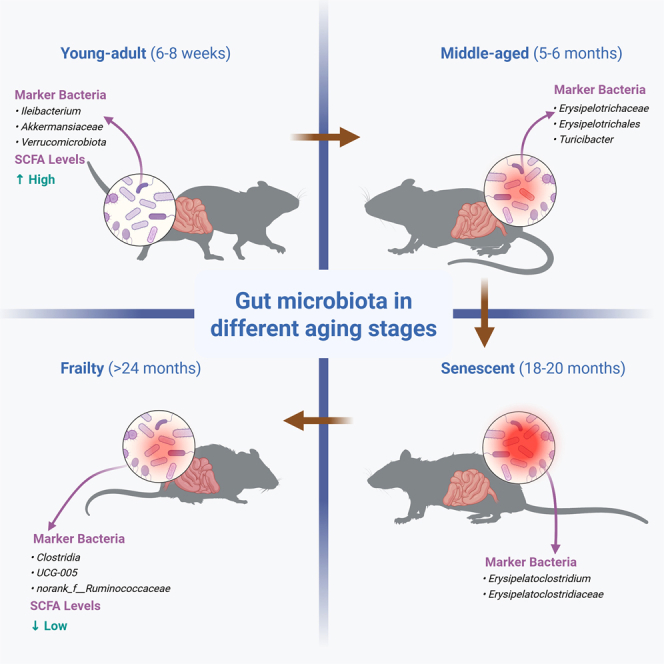

## Introduction

Aging is a progressive biological process characterized by gradual physiological decline and increased susceptibility to age-related diseases, including cardiovascular disorders, neurodegeneration, and metabolic dysfunction.^1^^,^^2^ With the global elderly population projected to exceed 2.1 billion by 2050, understanding aging mechanisms is increasingly urgent. One key mechanism involves the gut microbiota, which influences healthspan and lifespan through metabolic signaling, immune regulation, and neuro-immune interactions.^3^^,^^4^ Notably, an imbalanced gut microbial community, or dysbiosis, is frequently observed in aging and linked to frailty, chronic inflammation, and metabolic deterioration.^5^^,^^6^

Recent studies have identified age-associated microbial signatures, such as the decline of short-chain fatty acid (SCFA)-producing taxa like *Lachnospiraceae* and the enrichment of pro-inflammatory groups like *Enterobacteriaceae*.^7^^,^^8^ Additionally, reduced microbial diversity and SCFA production in aged mice are associated with increased intestinal permeability, systemic inflammation, and neurodegenerative progression.^9^^,^^10^^,^^11^ Although microbial metabolites impact systemic metabolism and cognitive function via the gut-brain and gut-liver axes,^12^^,^^13^^,^^14^ the functional implications of these microbial shifts across aging remain poorly understood.

Previous aging microbiome research often relies on cross-sectional designs, capturing only snapshots of microbial composition at specific time points.^14^^,^^15^ Variability in animal models, diets, and methods further contributes to inconsistent findings.^16^ While fecal microbiota transplantation (FMT) from young donors has shown promise in rejuvenating aging phenotypes in mice,^17^ the specific microbial and metabolic signatures of frailty remain underexplored.^18^

To address these gaps, we conducted a longitudinal study profiling gut microbiota dynamics across four aging stages in C57BL/6J mice: young-adult (6–8 weeks), middle-aged (5–6 months), senescent (18–20 months), and frailty (>24 months). Using 16S rRNA gene sequencing to assess microbial diversity and taxonomic composition, alongside targeted metabolomics to measure SCFA levels and PICRUSt2 for functional pathway prediction, our study aims to (1) map progressive microbial shifts across aging stages, (2) explore the relationship between microbiota changes and host metabolic functions, and (3) identify frailty-specific microbial signatures. Integrating microbiome and metabolome analyses, our findings offer new insights into the role of gut microbial alterations in aging and frailty, providing a basis for future microbiota-targeted interventions to promote healthy aging.

## Results

### Alpha diversity of gut microbiota across aging stages

**Figure 1:**
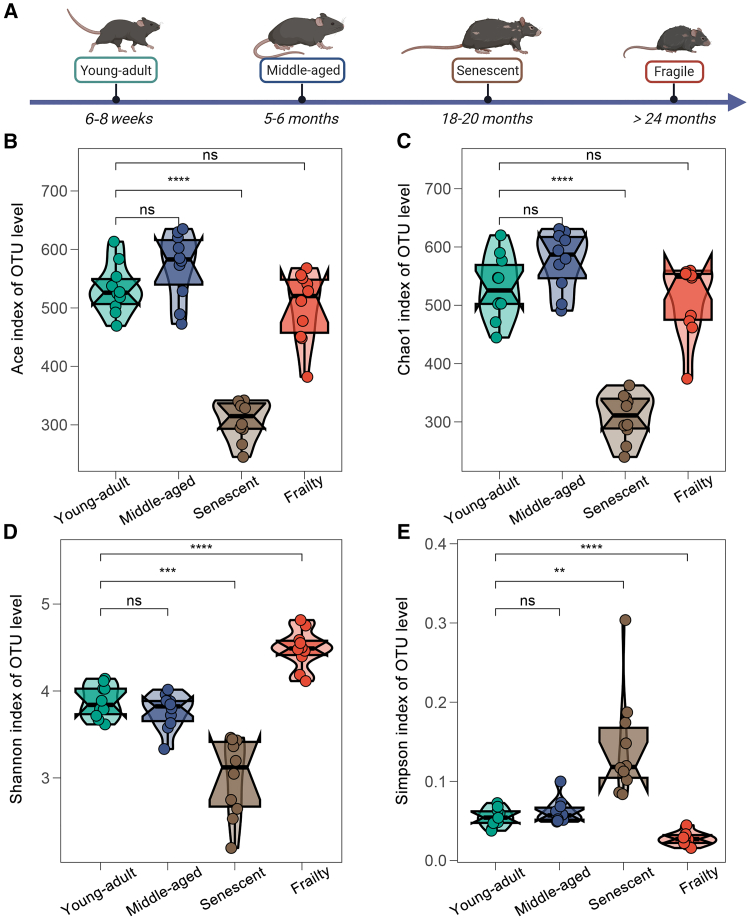
Impact of aging on gut microbiota alpha diversity in mice across four developmental stages (A) Schematic timeline of aging stages in C57BL/6J mice: young-adult (6–8 weeks), middle-aged (5–6 months), senescent (18–20 months), and frailty (>24 months), depicting gradual aging and physiological changes. (B–E) Alpha diversity indices (OTU-level) across four age groups (*n* = 10/group), shown as violin plots with medians: ACE index, (C) Chao1 index, (D) Shannon index, (E) Simpson index. Statistics: Wilcoxon rank-sum test vs. young-adult; significance: ns, not significant (*p* > 0.05); ∗∗*p* < 0.01; ∗∗∗*p* < 0.001; ∗∗∗∗*p* < 0.0001. Data are represented as mean ± SD.

To examine aging effects on gut microbiota, we assessed alpha diversity in fecal samples from C57BL/6J mice across four stages: young-adult (6–8 weeks), middle-aged (5–6 months), senescent (18–20 months), and frailty (>24 months) (Figure 1A). Microbial richness, measured by ACE and Chao1 indices, remained stable between young-adult and middle-aged mice (*p* > 0.05, Wilcoxon rank-sum test), indicating consistent species abundance in early aging (Figures 1B and 1C). In contrast, richness declined significantly in the Senescent group (ACE: *p* < 0.001; Chao1: *p* < 0.001), reflecting a loss of microbial taxa with aging. Notably, the Frailty group showed partial recovery, with ACE and Chao1 indices approaching young-adult levels (*p* > 0.05).

Microbial diversity, assessed by Shannon and Simpson indices, followed a distinct pattern. No significant changes occurred between young-adult and middle-aged groups (*p* > 0.05), but diversity dropped markedly in the senescent group (Shannon: *p* < 0.01; Simpson: *p* < 0.001), suggesting reduced evenness and increased taxon dominance (Figures 1D and 1E). The frailty group exhibited a significant diversity increase compared to the senescent group (Shannon: *p* < 0.001; Simpson: *p* < 0.001) and higher Simpson values than the young-adult group (*p* < 0.001), though Shannon differences were not significant (*p* > 0.05). This suggests a compensatory expansion of microbial heterogeneity in late-stage aging. Collectively, these findings indicate a non-linear trajectory of gut microbiota alpha diversity, with stability in early stages, a decline in senescence, and a rebound in frailty.

### Beta diversity and taxonomic composition changes across aging stages

**Figure 2:**
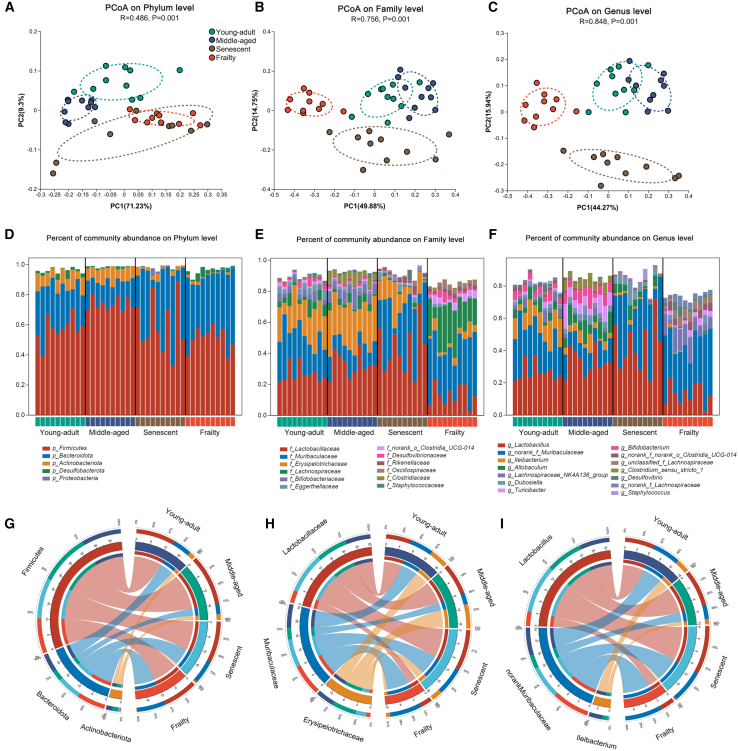
Effects of aging on gut microbiota beta diversity and taxonomic composition across four developmental stages in mice (A–C) PCoA based on Bray-Curtis dissimilarity at (A) phylum, (B) family, and (C) genus levels, showing microbial structure in fecal samples from young-adult, middle-aged, senescent, and frailty C57BL/6J mice (*n* = 10 per group). (D–F) Stacked bar plots displaying relative abundance of dominant taxa at (D) phylum, (E) family, and (F) genus levels across the four age groups. Each bar represents an individual sample, colored by taxa. (G–I) Circos plots visualizing distribution of the top three dominant taxa (by average relative abundance) at (G) phylum, (H) family, and (I) genus levels, illustrating compositional shifts across aging stages.

To investigate gut microbiota dynamics during aging, we assessed microbial community structure using principal coordinate analysis (PCoA) based on Bray-Curtis dissimilarity. Significant restructuring was observed across aging stages at the phylum (R^2^ = 0.486), family (R^2^ = 0.587), and genus levels (R^2^ = 0.485) (Figures 2A–2C; PERMANOVA, *p* < 0.001). At the phylum level, Frailty samples separated distinctly along PC1 (71.23% variance), whereas middle-aged and senescent groups showed partial overlap (Figure 2A). This divergence was less pronounced at the family (PC1: 48.88%) and genus levels (PC1: 42.77%), though frailty remained distinct from young-adult and senescent groups (Figures 2B and 2C), indicating profound aging-related shifts, especially in frailty.

We analyzed the relative abundance of dominant taxa (Figures 2D–2F). At the phylum level, *Firmicutes*, *Bacteroidota*, and *Actinobacteriota* were predominant. *Firmicutes* increased from young-adult to middle-aged (*p* < 0.01) before declining in senescent and frailty (*p* < 0.05 vs. young-adult). Conversely, *Bacteroidota* decreased from young-adult to middle-aged (*p* < 0.01) and then increased in senescent and frailty (*p* < 0.01 vs. young-adult). At the family level, *Lactobacillaceae* peaked in senescent mice (*p* < 0.01 vs. young-adult) but declined in frailty (*p* < 0.05). *Muribaculaceae* decreased from young-adult to middle-aged (*p* < 0.01) and increased in frailty (*p* < 0.05 vs. young-adult). *Erysipelotrichaceae* showed a progressive decline from young-adult to frailty (*p* < 0.01).

At the genus level, *Lactobacillus* mirrored its family trend, peaking in senescent mice (*p* < 0.01) before declining in frailty (*p* < 0.05). *Norank_f_Muribaculaceae* and *Ileibacterium* followed similar aging patterns, with *Ileibacterium* significantly reduced in frailty (*p* < 0.01). These taxonomic shifts were supported by Circos plots, highlighting distinct microbial composition patterns across aging stages (Figures 2G–2I). Collectively, these findings reveal significant age-associated microbial restructuring, with frailty as a key transition point in gut microbiota composition.

### Discriminative microbial taxa associated with aging stages

To identify key microbial taxa driving age-associated differences, we performed linear discriminant analysis effect size (LEfSe) analysis, comparing young-adult mice with middle-aged, senescent, and frailty groups. Taxa with a linear discriminant analysis (LDA) score >4.0 and *p* < 0.05 (Wilcoxon rank-sum test) were considered significantly discriminative.

**Figure 3:**
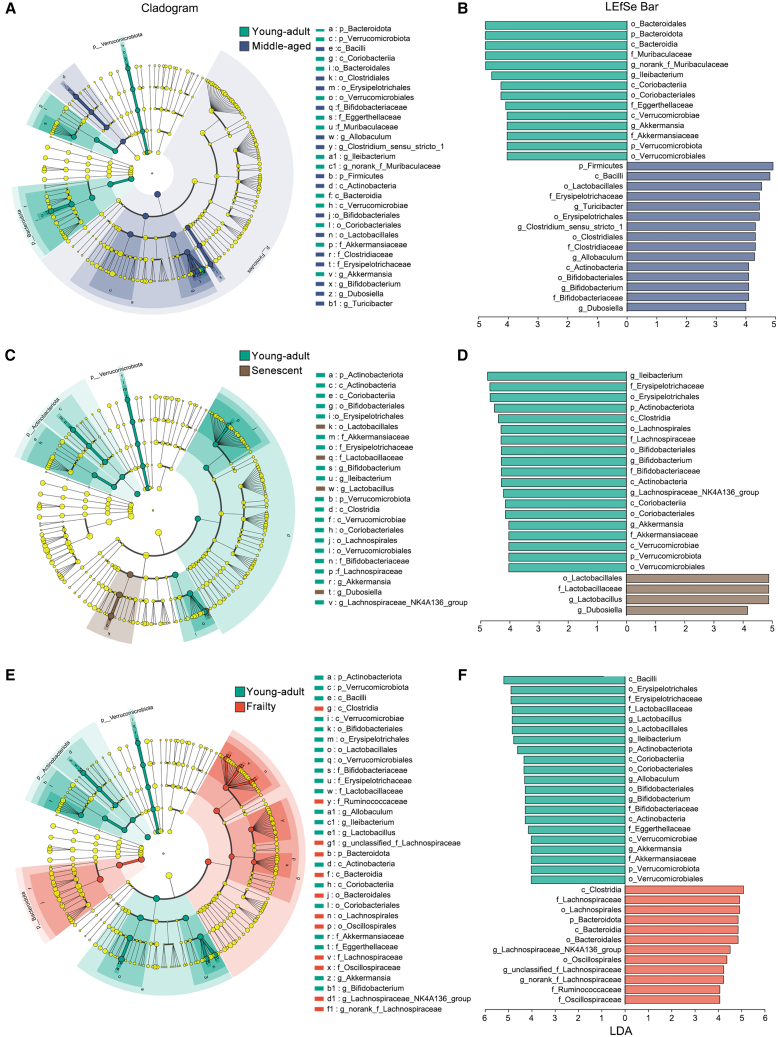
Identification of discriminative microbial taxa across aging stages in mice using LEfSe analysis (A and B) Comparison between middle-aged and young-adult mice. (A) Cladogram representing taxonomic hierarchy of differentially abundant taxa. The color-shaded areas highlight enriched taxa in the respective groups. (B) LEfSe bar plot showing LDA scores of differentially abundant taxa (LDA score >4.0, *p* < 0.05, Wilcoxon rank-sum test). (C and D) Comparison between senescent and young-adult mice: (C) Cladogram and (D) LEfSe bar plot indicating microbial taxa enriched in senescent and young-adult groups. (E and F) Comparison between frailty and young-adult mice. (E) Cladogram and (F) LEfSe bar plot illustrating frailty-associated microbial taxa with distinct enrichment patterns.

In middle-aged mice, *Bacteroidota*-related taxa were depleted, whereas *Firmicutes* and *Actinobacteriota* taxa, including *Dubosiella*, were enriched (Figures 3A and 3B). In the senescent group, *Ileibacterium* and *Erysipelotrichaceae* showed notable reductions (LDA >4.0, *p* < 0.01), consistent with the alpha diversity decline (Figure 1), whereas *Lactobacillus* was significantly enriched (Figures 3C and 3D). In the frailty group, *Firmicutes* taxa, such as *Bacilli* and *Erysipelotrichaceae*, were significantly depleted (LDA >4.0, *p* < 0.01), whereas *Bacteroidota* and *Firmicutes* taxa, including *Oscillospiraceae* and *Lachnospiraceae_NK4A136_group*, were enriched (LDA >4.0, *p* < 0.01) (Figures 3E and 3F).

The enrichment of SCFA-producing taxa in Frailty suggests a potential link to the observed diversity rebound (Figure 1). These results indicate progressive shifts in gut microbiota composition with aging, with distinct microbial signatures in the frailty stage.

### Co-occurrence networks reveal increased microbial interactions with aging

**Figure 4:**
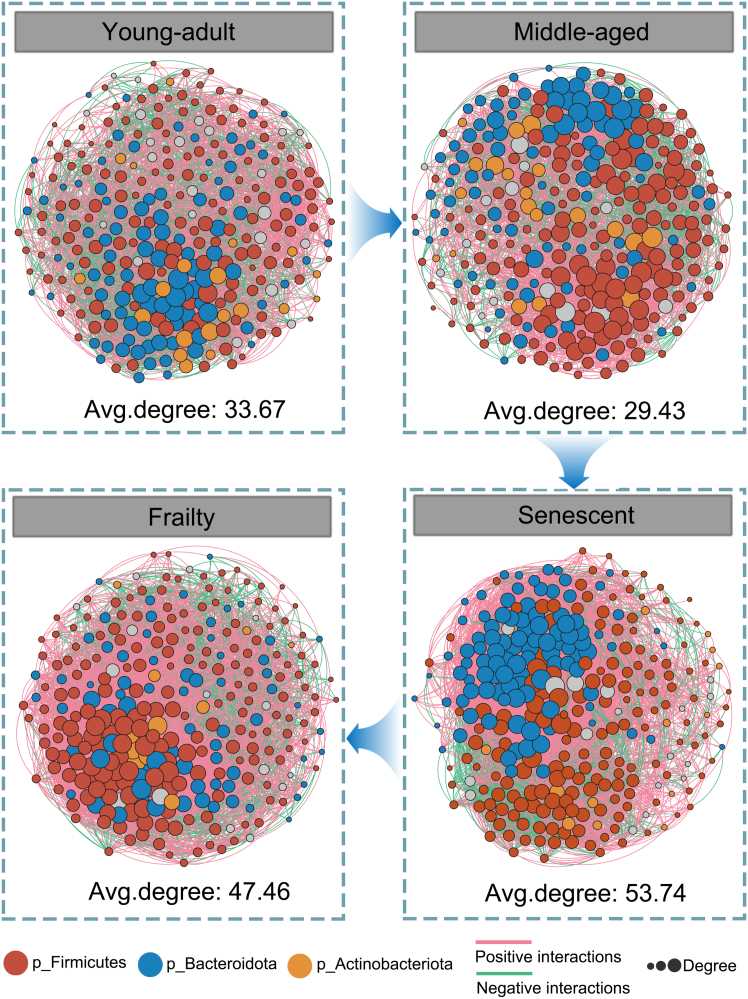
Co-occurrence networks of gut microbiota across aging stages in mice Co-occurrence networks were constructed for gut microbiota of young-adult, middle-aged, senescent, and frailty C57BL/6J mice using the top 300 abundant OTUs per group. Spearman correlation analysis (absolute coefficient >0.5, *p* < 0.05) defined microbial associations. Nodes represent OTUs, colored by phylum: *Firmicutes* (red), *Bacteroidota* (blue), *Actinobacteriota* (orange). Edges indicate significant interactions: positive correlations (green) and negative correlations (red). Node size reflects degree centrality, with larger nodes showing higher connectivity. Average degree (Avg. degree) per network reflects overall complexity and connectivity within each age group.

To investigate aging impacts on microbial interactions, we constructed co-occurrence networks for the top 300 abundant OTUs in each group using Spearman correlation analysis (absolute coefficient >0.5, *p* < 0.05). Building on discriminative taxa identification, this analysis uncovered potential synergistic or antagonistic relationships shaping community dynamics (Figure 4).

The young-adult network showed a stable interaction pattern with an average degree of 33.67, whereas the middle-aged network had reduced connectivity (29.43), suggesting fewer microbial interactions in early aging. In contrast, the senescent network exhibited increased complexity, with an average degree of 53.74, significantly higher than young-adult (*p* < 0.01, Wilcoxon rank-sum test). The frailty network maintained high connectivity (47.46), also significantly elevated compared to young-adult (*p* < 0.05).

This analysis indicates a progressive shift in microbial network structure across aging stages, with greater connectivity in senescent and frailty stages than in earlier stages, suggesting altered microbial interactions and potential dysbiosis linked to aging.

### Functional profiling of gut microbiota reveals age-associated metabolic shifts

**Figure 5:**
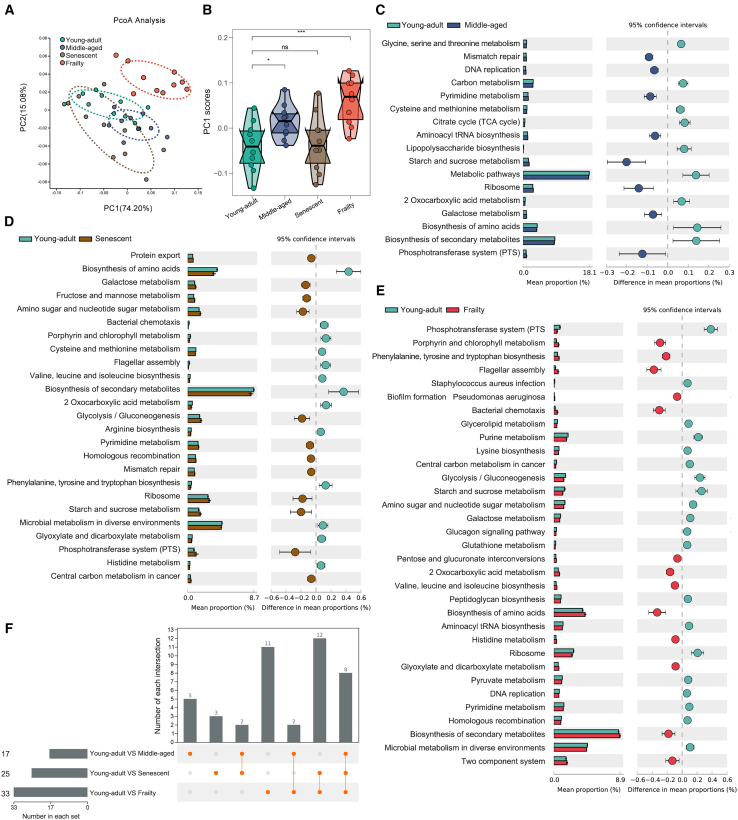
PICRUSt2-predicted functional pathways of gut microbiota across aging stages in mice (A) PCoA of KEGG level 3 pathways based on Bray-Curtis dissimilarity, showing functional shifts in gut microbiota across aging stages in C57BL/6J mice. (B) Boxplot of PC1 scores comparing pathway compositions among groups. Statistical significance determined by Wilcoxon rank-sum test (ns, not significant, *p* > 0.05; ∗*p* < 0.05; ∗∗*p* < 0.01 vs. Young-adult). (C–E) STAMP analysis of differentially abundant KEGG pathways between young-adult and (C) middle-aged, (D) senescent, and (E) frailty groups (Welch’s t test, *p* < 0.05). Bars represent mean proportions with 95% confidence intervals. (F) Bar plot summarizing the number of differentially enriched pathways across comparisons: young-adult vs. middle-aged, 17 pathways; young-adult vs. senescent, 25 pathways; young-adult vs. frailty, 33 pathways. Data are represented as mean ± SD.

To assess functional consequences of microbial taxonomic shifts and increased interaction complexity across aging stages, we used PICRUSt2 to predict gut microbiota functional profiles from 16S rRNA sequencing data. PCoA of KEGG level 3 pathways, based on Bray-Curtis dissimilarity, showed significant functional separation across groups, with PC1 and PC2 explaining 74.20% and 15.08% of the variance, respectively. The frailty group clustered distinctly along PC1 (Figure 5A). Boxplot analysis of PC1 scores confirmed a significantly altered functional profile in frailty compared to young-adult (*p* < 0.01, Wilcoxon rank-sum test), with middle-aged and senescent groups showing moderate shifts (*p* < 0.05) (Figure 5B).

STAMP analysis identified differentially abundant KEGG pathways between young-adult and other groups (*p* < 0.05, Welch’s t test). Middle-aged mice showed enrichment in metabolic pathways, such as glycine, serine, and threonine metabolism (Figure 5C), whereas senescent mice exhibited upregulation in pathways including protein export and fructose/mannose metabolism (Figure 5D). Frailty displayed the most pronounced alterations, with unique pathways potentially linked to inflammation and oxidative stress, such as *Staphylococcus aureus* infection and *Pseudomonas aeruginosa* biofilm formation (Figure 5E).

An upset plot highlighted pathway overlap and uniqueness, showing frailty with the highest number of distinct pathways (33), followed by senescent (25) and middle-aged (17) (Figure 5F). These results indicate a progressive shift in gut microbiota functional pathways with aging, with frailty exhibiting the most marked changes, particularly in metabolic and biosynthetic pathways.

### Correlation between gut microbiota and KEGG level 2 pathways in frailty-stage mice

**Figure 6:**
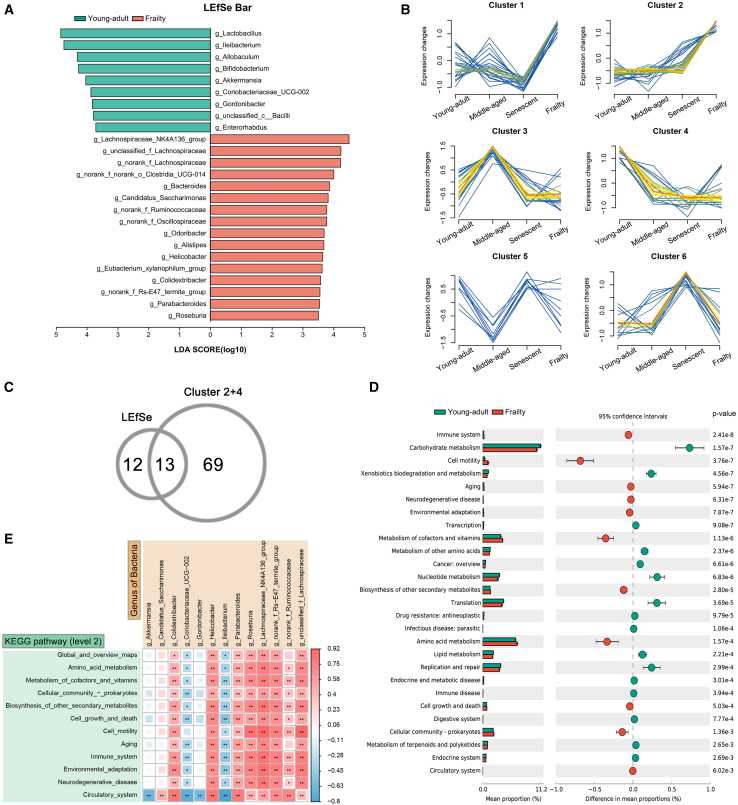
Correlation analysis of gut microbiota and KEGG level 2 functional pathways in frailty-stage mice (A) LEfSe analysis identifying genera differing between young-adult (green) and frailty (red) groups (LDA score >3.5, *p* < 0.05). (B) Longitudinal abundance trends of genera across young-adult, middle-aged, senescent, and frailty stages, clustered into six patterns. Clusters 2 and 4 selected for further analysis. (C) Venn diagram showing 13 key genera overlapping between clusters 2 and 4 and LEfSe-identified genera. (D) STAMP analysis of KEGG level 2 pathways differing between young-adult (green) and frailty (red) (Welch’s t test, *p* < 0.01). Bars show mean proportions with 95% confidence intervals. (E) Pearson correlation heatmap of 13 frailty-associated genera and KEGG level 2 pathways. Red for positive, blue for negative correlations (∗*p* < 0.05; ∗∗*p* < 0.01). Data are represented as mean ± SD.

To identify genera linked to frailty-specific microbial shifts, we conducted LEfSe analysis to determine differentially abundant genera between young-adult and frailty groups (Figure 6A). We then examined abundance trends of all genera across the four aging stages, clustering them into six distinct patterns based on temporal dynamics (Figure 6B). Clusters 2 and 4 were selected for their significant aging-related trends: cluster 2 showed sharp upregulation in frailty, whereas cluster 4 exhibited an early decline followed by stabilization. Intersecting these with LEfSe-identified genera yielded 13 key genera, including *Akkermansia* and *Lachnospiraceae_NK4A136_group* (Figure 6C).

To explore functional implications, we correlated these genera with KEGG level 2 pathways (Figures 6D and 6E). *Akkermansia* and *Lachnospiraceae_NK4A136_group* showed negative correlations with carbohydrate metabolism (*p* < 0.01), whereas *Colidextribacter* and *Helicobacter* exhibited positive correlations with immune system pathways (*p* < 0.05), suggesting involvement in frailty-associated inflammation. These findings indicate that gut microbiota alterations in frailty are linked to host metabolic and immune dysregulation, highlighting their role in aging.

### Reduced SCFA levels in frailty-stage mice and their correlations with differential genera

**Figure 7:**
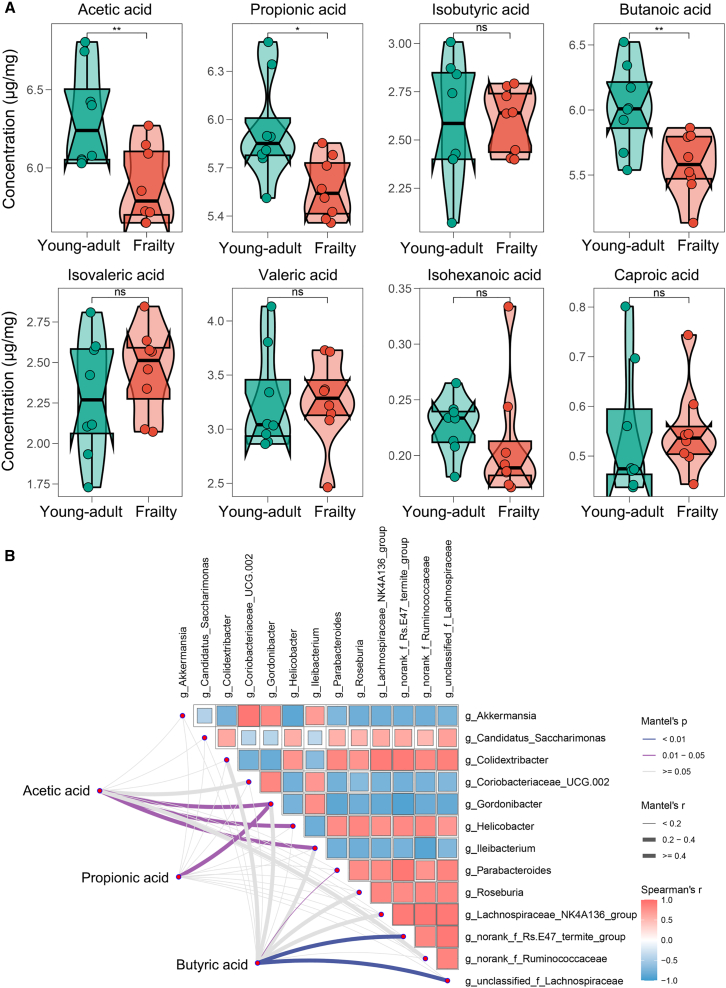
Short-chain fatty acid (SCFA) levels and their correlation with gut microbiota in frailty-stage mice (A) Violin plots showing concentrations of eight SCFAs in fecal samples from young-adult and frailty groups. Statistical analysis by one-way ANOVA with Tukey’s post-hoc test (∗*p* < 0.05; ∗∗*p* < 0.01). (B) Pearson correlation heatmap of SCFA levels and 13 frailty-associated genera. Red for positive, blue for negative correlations; intensity reflects strength. Significant correlations marked (∗*p* < 0.05; ∗∗*p* < 0.01). Data are represented as mean ± SD.

To investigate frailty’s impact on short-chain fatty acid (SCFA) production, we quantified fecal SCFA levels in young-adult and frailty groups using targeted metabolomics. Given the downregulation of carbohydrate metabolism pathways in frailty mice, critical for SCFA biosynthesis, we hypothesized a reduction in SCFA levels. Among the eight SCFAs analyzed, acetic, propanoic, and butanoic acids were significantly reduced in frailty compared to young-adult (*p* < 0.01, one-way ANOVA with Tukey’s post-hoc test), whereas the remaining SCFAs showed no significant differences (*p* > 0.05) (Figure 7A). These declines indicate notable depletion of key microbial metabolites in frailty. Detailed SCFA concentrations for each sample are provided in Table S1.

To assess microbial contributions to SCFA reductions, we performed Pearson correlation analysis between the 13 frailty-associated genera (Figure 6B) and the three altered SCFAs, validated by Mantel’s test (*p* < 0.01) and Spearman correlation coefficients. Acetic acid correlated with several genera, with notable significance for specific taxa, whereas propanoic acid showed positive correlations with *Allobaculum* and *Gordonibacter* (*p* < 0.05). Butanoic acid, a key gut-barrier-protecting metabolite, was strongly positively correlated with SCFA-producing taxa, including *Lachnospiraceae_NK4A136_group*, *Lactobacillus*, and *Bifidobacterium* (*p* < 0.01). Conversely, *Alistipes* exhibited a negative correlation with butanoic acid (*p* < 0.05), suggesting a potential inhibitory effect via competitive interactions.

These results indicate that gut microbiota compositional changes in frailty-stage mice are linked to disrupted SCFA metabolism, potentially contributing to host metabolic dysfunction.

## Discussion

In this study, we present a comprehensive analysis of gut microbiota dynamics across four aging stages in *C57BL/6J* mice, revealing a complex, non-linear trajectory in microbial diversity, taxonomic composition, inter-microbial interactions, functional pathway alterations, and metabolic profiles. Our findings highlight that age-related microbial changes follow a biphasic pattern, reflecting both adaptive and pathological processes, particularly in frailty. Frailty status in mice aged >24 months was defined using a modified Clinical Frailty Index (CFI), assessing parameters such as body weight loss (>10%), poor coat condition, kyphosis, reduced locomotor activity, and ocular signs.^19^ However, we acknowledge that a more neutral term like “advanced senescence” could reduce ambiguity, and future studies incorporating quantitative metrics like grip strength could further validate this classification. Additionally, while health monitoring ensured no overt pathologies (e.g., tumors and infections), systematic histopathological assessments were not performed, leaving open the possibility that the observed microbial shifts reflect underlying aging-related conditions, such as chronic inflammation or intestinal barrier alterations. Future studies with comprehensive pathological analyses will clarify whether microbiota restructuring represents adaptive remodeling or pathology-driven dysbiosis.

### Non-linear changes in microbial diversity and taxonomic restructuring

A striking observation is the biphasic shift in alpha diversity. Microbial richness, measured by ACE and Chao1 indices, declined significantly in the senescent group but partially rebounded in frailty. This rebound likely reflects a “pathological compensation” or microbial community rearrangement rather than a beneficial restoration, as it coincided with pro-inflammatory taxonomic shifts (e.g., *Bacteroidota* enrichment and *Firmicutes* depletion) and SCFA depletion. A similar trend of increased diversity has been reported in human long-living populations (aged 90+ years), alongside microbial rearrangement involving taxa like *Bifidobacterium*.^20^^,^^21^ However, frailty is generally associated with decreased diversity, and unhealthy long-living individuals show lower diversity than healthy ones,^21^^,^^22^ supporting the notion of a maladaptive response. Future studies integrating host health indicators, such as inflammatory cytokine levels and behavioral tests, could clarify the functional significance of this rebound. Frailty-stage microbiota showed enrichment of *Bacteroidota* and depletion of *Firmicutes*, trends observed in frail elderly humans.^8^^,^^14^ This suggests that increased diversity may not reflect improved gut health but a restructured community with enhanced taxon interactions, potentially driving a pro-inflammatory state.^5^

### Altered microbial interactions and functional shifts

Co-occurrence network analysis revealed increased microbial interactions in senescent and frailty stages compared to early adulthood. Enhanced connectivity and clustering in frailty suggest that altered inter-species relationships may drive dysbiosis.^23^ However, the modest sample size (*n* = 10 per group) may limit the statistical robustness of inferred associations. We applied stringent thresholds to enhance reliability, but future studies with larger cohorts and advanced network inference methods will be necessary to validate these observations. This restructuring could promote pro-inflammatory taxa expansion while reducing beneficial SCFA-producing microbes, notably within *Firmicutes*.^23^ PICRUSt2 predictions support this, showing upregulated immune and aging-related pathways and downregulated carbohydrate metabolism in frailty.^24^^,^^25^ These functional shifts may result from or contribute to host immunometabolic alterations, reflecting a bidirectional interplay. Systemic immune changes, such as elevated interleukin-6 (IL-6) or tumor necrosis factor alpha (TNF-α), and altered metabolic hormones like insulin and leptin, likely shape microbial ecology in frailty. Our observational design captures associations rather than causality, and future studies using FMT or antibiotic-mediated depletion followed by recolonization could clarify the microbiome’s role in driving these changes. These functional changes may underlie metabolic disruptions, particularly the depletion of SCFAs like butanoic acid, a key anti-inflammatory and gut-barrier-protective metabolite,^26^^,^^27^^,^^28^ observed in targeted metabolomics.

### Implications for frailty and metabolic dysfunction

The convergence of taxonomic shifts, altered networks, and functional remodeling suggests a mechanistic link between gut dysbiosis and frailty. Enrichment of taxa like *Lachnospiraceae_NK4A136_group* in frailty, strongly correlated with butanoic acid levels, indicates a compensatory yet insufficient response to maintain gut homeostasis.^29^ The increase in *Lachnospiraceae_NK4A136_group*, despite declining SCFA levels, suggests a compensatory but ineffective response, potentially due to reduced substrate availability (e.g., dietary fiber), disrupted microbial cross-feeding, or increased host SCFA uptake.^10^ Conversely, the negative correlation with *Alistipes* suggests competitive interactions further depleting SCFAs.^30^ Genus-level analysis of *Lachnospiraceae* and *Alistipes* limits functional resolution, as these genera encompass species with diverse roles (e.g., *Roseburia* spp. as butyrate producers).^31^ Future studies using shotgun metagenomics or metatranscriptomics could validate the roles of specific taxa in SCFA production and host responses during aging. These gut metabolic changes may exacerbate systemic inflammation and metabolic dysfunction, aligning with evidence linking microbiota to age-related frailty.^17^^,^^32^^,^^33^

### Integrating current findings with existing literature

Our longitudinal study builds on cross-sectional research, highlighting the non-linear nature of microbial transitions in aging.^15^ While prior studies noted losses of SCFA producers and gains in pro-inflammatory taxa,^6^ our data show these shifts are complex and biphasic. The diversity rebound in frailty, despite an adverse taxonomic profile, suggests adaptive responses to physiological decline.^20^ However, this may also perpetuate inflammation, reinforcing frailty.^23^ This interplay between microbial diversity, metabolic function, and host inflammation aligns with recent findings positioning gut microbiota as both markers and mediators of aging and frailty.^2^^,^^24^^,^^34^ Our study used only male mice to minimize hormonal variability,^10^ but this limits generalizability, as sex differences can influence microbial changes. Future studies including both sexes could elucidate sex-specific patterns in frailty.

### Limitations of the study

Our study has limitations. 16S rRNA gene sequencing, while effective for taxonomic profiling, limits species-level resolution and functional insights compared to metagenomics. Our sequencing depth analysis confirmed sufficient read counts for taxonomic resolution, with raw read counts ranging from 33,249 to 74,852 and filtered read counts from approximately 26,570 to 56,861 per sample (Table S2), ensuring reliable microbial profiling. Mice were cohoused within age groups (3–5 per cage) to standardize conditions, which may reduce microbial variation.^35^^,^^36^^,^^37^ However, cage density and environmental factors could still influence microbiome composition, and future studies with individual housing could disentangle age-driven versus environmental effects.^38^^,^^39^

Additionally, while fecal samples were snap-frozen within 5 min of collection to minimize microbial shifts,^40^ further standardization of collection timing could eliminate residual variability. The longitudinal design offers snapshots of microbial dynamics, but more frequent sampling and multi-omics (e.g., metatranscriptomics, metabolomics) are needed to capture temporal complexity. Our targeted metabolomics focused on SCFAs, but frailty involves broader metabolic alterations, including bile acids and branched-chain amino acids.^26^ PICRUSt2 analysis suggested disrupted carbohydrate metabolism in frailty; future studies profiling carbohydrate intermediates (e.g., glucose and lactate) using untargeted metabolomics could provide deeper mechanistic insights. Additionally, translating murine findings to human aging requires caution due to species differences. Nonetheless, our results provide a foundation for future microbiota-targeted interventions to restore balance and mitigate frailty-associated decline.

In conclusion, our study demonstrates that gut microbiota undergo significant, non-linear changes during aging, with taxonomic and functional shifts closely tied to frailty. These findings underscore gut dysbiosis as a key factor in aging pathophysiology and suggest that microbiota interventions (e.g., prebiotics, probiotics, and fecal microbiota transplantation) may promote healthy aging and reduce frailty-associated decline. Further multi-omics studies and clinical validation are essential to translate these insights into effective therapies.

## Resource availability

### Lead contact

Further information and requests for resources and reagents should be directed to and will be fulfilled by the lead contact, Zhijie Li (tzhijieli@jnu.edu.cn).

### Materials availability

This study did not generate new unique reagents.

### Data and code availability

• 16S rRNA gene sequencing data generated in this study has been deposited at the NCBI Sequence Read Archive (SRA) and is publicly available as of the date of publication. Accession numbers are listed in the key resources table under BioProject PRJNA1003690.
• This paper does not report original code. All analyses were performed using standardized pipelines on the Majorbio Cloud Platform (https://cloud.majorbio.com/), as detailed in the STAR Methods (quantification and statistical analysis).
• Any additional information required to reanalyze the data reported in this paper is available from the lead contact upon request.

## Acknowledgments

This work was supported by the Basic Science Project of Henan Eye Institute/Henan Eye Hospital (Grant No. 21JCZD001 to Z.L.) and the Doctoral Startup Fund of the Affiliated Hospital of Shandong Second Medical University (Grant No. 2024BSQD05 to X.J.).

## Author contributions

X.J. and Z.L. conceived and designed the study. X.J. performed the animal experiments and collected the fecal samples. X.J. and X.L. conducted the 16S rRNA sequencing and metabolomics analyses. H.L., T.W., S.W., H.F., and L.W. performed the bioinformatics and statistical analyses. A.D. and Z.L. supervised the project. X.J. and Z.L. provided funding. X.J. and Z.L. wrote the manuscript with input from all authors. All authors reviewed and approved the final manuscript.

## Declaration of interests

The authors declare no competing interests.

## STAR★Methods

### Key resources table

**Table:** 

REAGENT or RESOURCE	SOURCE	IDENTIFIER
**Biological samples**		

Mouse fecal samples from C57BL/6J mice	This paper	N/A

**Critical commercial assays**		

E.Z.N.A.® Soil DNA Kit	Omega Bio-Tek	Cat#D5625
2× Phusion High-Fidelity PCR Master Mix	New England Biolabs	Cat#M0531S
AxyPrep DNA Gel Extraction Kit	Axygen Biosciences	Cat#AP-GX-250

**Deposited data**		

16S rRNA sequencing data	NCBI Sequence Read Archive (SRA)	BioProject PRJNA1003690

**Experimental models: Organisms/strains**		

Mouse: C57BL/6J: Wild-type	GemPharmatech Co., Ltd.	RRID:IMSR_JAX:000664

**Oligonucleotides**		

Primer 338F (5′-ACTCCTACGGGAGGCAGCAG-3′)	Shanghai Meiji Biological Technology	N/A
Primer 806R (5′-GGACTACHVGGGTWTCTAAT-3′)	Shanghai Meiji Biological Technology	N/A

**Software and algorithms**		

Trimmomatic, version 0.39	N/A	http://www.usadellab.org/cms/?page=trimmomatic; RRID:SCR_011848
FLASH, version 1.2.11	N/A	http://ccb.jhu.edu/software/FLASH/; RRID:SCR_005531
UPARSE, version 7.0.1090	N/A	http://drive5.com/uparse/; RRID:SCR_005020
RDP Classifier, version 2.11	N/A	https://rdp.cme.msu.edu/classifier/; RRID:SCR_001591
Mothur, version 1.31.2	N/A	https://mothur.org/; RRID:SCR_011947
QIIME2, version 2022.2	N/A	https://qiime2.org/; RRID:SCR_021258
PICRUSt2, version 2.2.0	N/A	https://github.com/picrust/picrust2; RRID:SCR_022646
STAMP, version 2.1.3	N/A	https://beikolab.cs.dal.ca/software/STAMP; RRID:SCR_018637
Majorbio Cloud Platform	N/A	https://cloud.majorbio.com/
R, version 4.4.3	N/A	https://www.r-project.org/; RRID:SCR_001905
SILVA, version 138	N/A	https://www.arb-silva.de/; RRID:SCR_006568

**Other**		

Agilent 7890B GC-MS System	Agilent Technologies	N/A
4-Methylvaleric acid (internal standard)	Sigma-Aldrich	Cat# 277827; N/A
Methyl *tert*-butyl ether (MTBE)	Sigma-Aldrich	Cat#306975; N/A

### Experimental model and study participant details

#### Animal experiments

All animal procedures were conducted in accordance with the ARVO Statement for the Use of Animals in Vision and Ophthalmology Research and approved by the Henan Provincial People’s Hospital’s Institutional Animal Care and Use Committee. Male C57BL/6J mice were obtained from GemPharmatech Co., Ltd. (RRID:IMSR_JAX:000664) to minimize variability due to hormonal fluctuations in females^10^ and housed under specific pathogen-free (SPF) conditions at 22 ± 1°C, 60 ± 5% relative humidity, and a 12-h light/12-h dark cycle. Mice had *ad libitum* access to a standard chow diet and sterile water, and were housed with corncob bedding (Shandong Pengyue Experimental Animal Technology Co., Ltd.).

Four aging stages were defined based on the C57BL/6J mouse life cycle and physiological changes associated with aging^19^^,^^41^: Young-adult (6–8 weeks, *n* = 10), representing early adulthood with stable growth and development; Middle-aged (6–8 months, *n* = 10), displaying no overt aging phenotypes and marking the onset of early aging signs such as metabolic changes; Senescent (18–20 months, *n* = 10), characterized by significant physiological decline including weight reduction, fur graying, and decreased activity; and Frailty (>24 months, *n* = 10), reflecting extreme aging with pronounced frailty traits as evidenced by advanced age, typical survival decline, and phenotypic assessments (e.g., body weight loss >10%, poor coat condition, kyphosis), with mice exhibiting three or more of these criteria classified as frail. Systemic immunological markers (e.g., IL-6, TNF-α) were not measured to validate age-stage classifications. Health monitoring ensured no overt pathological conditions (e.g., tumors, infections) in the Frailty group, with classification based on phenotypic assessments. Mice within each age group were cohoused (3–5 per cage) to standardize microbial exposure,^42^ with littermates randomly assigned to groups. Different age groups were housed separately to avoid cross-contamination. At the end of the experiment, mice were euthanized via isoflurane overdose followed by cervical dislocation, with all efforts made to minimize animal suffering. Sex-specific differences were not assessed due to the use of male mice to control for variability.

### Method details

#### Fecal sample collection

Fecal samples were collected from each mouse at the designated aging stage under sterile conditions. To minimize contamination, mice were individually housed in sterile cages for 2 h, after which freshly voided fecal pellets were aseptically transferred into sterile 1.5-mL microcentrifuge tubes. Samples were immediately snap-frozen in liquid nitrogen within 5 min of collection and stored at −80°C until analysis. A total of 40 samples (10 per group) were collected for downstream microbiome and metabolomics analyses.

#### DNA extraction

Total genomic DNA was extracted from fecal samples (∼200 mg per sample) using the E.Z.N.A. Soil DNA Kit (Omega Bio-Tek, Cat#D5625) per the manufacturer’s instructions, consistent with our previous studies.^43^^,^^44^^,^^45^ DNA concentration and purity were assessed with a NanoDrop 2000 spectrophotometer (Thermo Scientific), and integrity was verified via 1% agarose gel electrophoresis. Negative controls (blank extractions) were included to monitor contamination.

#### 16S rRNA gene sequencing

The V3-V4 hypervariable regions of the bacterial 16S rRNA gene were amplified using primers 338F (5′-ACTCCTACGGGAGGCAGCAG-3′) and 806R (5′-GGACTACHVGGGTWTCTAAT-3′), provided by Shanghai Meiji Biological Technology, in a GeneAmp 9700 PCR system (Applied Biosystems). PCR reactions were performed in triplicate in 25-μL mixtures containing 12.5 μL of 2× Phusion High-Fidelity PCR Master Mix (New England Biolabs, Cat#M0531S), 0.5 μM of each primer, and 10 ng of template DNA. Cycling conditions were: initial denaturation at 95°C for 3 min; 27 cycles of 95°C for 30 s, 55°C for 30 s, and 72°C for 45 s; and a final extension at 72°C for 10 min. Amplicons were purified with the AxyPrep DNA Gel Extraction Kit (Axygen Biosciences, Cat#AP-GX-250), quantified using an FTC-3000TM real-time PCR system (Funglyn Biotech), pooled in equimolar amounts, and sequenced on an Illumina MiSeq platform (Illumina) with 2 × 300 bp paired-end sequencing. Raw and post-QC sequencing depths for all 40 samples are provided in Table S2, confirming sufficient read counts for taxonomic resolution.

#### Bioinformatics analysis

Raw FASTQ files were quality-filtered using Trimmomatic (v0.39, RRID:SCR_011848) with a 4-bp sliding window and minimum quality score of 20, followed by sequence merging with FLASH (v1.2.11; Magoč and Salzberg, RRID:SCR_005531). Operational taxonomic units (OTUs) were clustered at 97% similarity using UPARSE (v7.0.1090, RRID:SCR_005020) with chimera removal. Taxonomic classification was performed using the RDP Classifier (v2.11, RRID:SCR_001591) against the SILVA 138 16S rRNA database^46^ (RRID:SCR_006568) with a confidence threshold ≥0.7. Alpha diversity indices (Chao1, ACE, Shannon, Simpson) were calculated using Mothur (v1.31.2, RRID:SCR_011947), with rarefaction curves to assess sequencing depth. Beta diversity was evaluated via Principal Coordinates Analysis (PCoA) based on Bray-Curtis distances at the OTU level using QIIME2 (v2022.2, RRID:SCR_021258). Group differences were assessed by the Wilcoxon rank-sum test. Linear discriminant analysis effect size (LEfSe) identified discriminative taxa (LDA score >3.5, *p* < 0.05). Microbial co-occurrence networks were constructed using Spearman correlation (absolute coefficient >0.5, *p* < 0.05) based on the top 300 abundant OTUs per group. Functional profiles were predicted with PICRUSt2 (v2.2.0, RRID:SCR_022646) and annotated using the Kyoto Encyclopedia of Genes and Genomes (KEGG) database. Significant functional differences were identified using STAMP (v2.1.3, RRID:SCR_018637) with Welch’s t-test (*p* < 0.05). Analyses were performed on the Majorbio Cloud Platform (https://cloud.majorbio.com/).^47^

#### Targeted metabolomics of short-chain fatty acids (SCFAs)

Fecal SCFAs (acetic, propanoic, butanoic, isobutyric, isovaleric, valeric, isohexanoic, and hexanoic acids) were quantified using gas chromatography-mass spectrometry (GC-MS). Briefly, 50 mg of fecal sample was homogenized in 500 μL of 0.5% phosphoric acid, spiked with 50 μL of 100 μg/mL 4-methylvaleric acid (internal standard; Sigma-Aldrich, Cat# 277827), and extracted with 500 μL of methyl *tert*-butyl ether (MTBE; Sigma-Aldrich, Cat#306975). After centrifugation at 12,000 × g for 10 min at 4°C, the supernatant was analyzed using an Agilent 7890B GC system coupled with a 5977A mass selective detector (Agilent Technologies). SCFAs were separated on an HP-FFAP column (30 m × 0.25 mm × 0.25 μm) with helium as the carrier gas (1 mL/min). Calibration curves were generated using SCFA standards (Sigma-Aldrich), and concentrations were normalized to sample weight.

#### Correlation analysis and validation of Microbial-SCFA relationships

Associations between gut microbiota and SCFAs were assessed via Pearson correlation analysis between SCFA concentrations and differentially abundant genera. Validation was performed using Mantel’s test, correlating Spearman-based microbial compositional distances with Euclidean SCFA concentration distances (999 permutations), ensuring robust microbial-SCFA relationships.

### Quantification and statistical analysis

#### Statistical analysis

Data are presented as means ± standard deviation (SD). Normality was assessed with the Shapiro-Wilk test. Group comparisons for alpha diversity, taxonomic abundance, and SCFA levels used one-way ANOVA with Tukey’s post-hoc test for parametric data or Wilcoxon rank-sum test for non-parametric data. SCFA functional differences were assessed using Welch’s t-test in STAMP (v2.1.3). Correlations between taxa and SCFAs used Pearson’s correlation coefficient. Microbial co-occurrence networks were constructed with Spearman correlation (absolute coefficient >0.5, *p* < 0.05). LEfSe analysis used a significance threshold of LDA score >3.5 and *p* < 0.05. Beta diversity group differences were assessed via Wilcoxon rank-sum test. All statistical parameters (e.g., exact value of n, means, SD, *p*-values) are reported in the figures, figure legends, and results section. No data or subjects were excluded; all analyses assumed normality unless non-parametric tests were applied. Statistical analyses were conducted in R (v4.4.3, RRID:SCR_001905).

### Additional resources

This study has not generated or contributed to a new website/forum, nor is it part of a clinical trial. All analyses were performed on the Majorbio Cloud Platform, a publicly accessible bioinformatics platform (https://cloud.majorbio.com/), which provides standardized pipelines for 16S rRNA sequencing data analysis.
