# Supplementary material for: Progressive gut microbiota shifts and functional alterations across aging stages and frailty in mice
Source: iScience. 2025 Jun 23;28(7):112985. doi: 10.1016/j.isci.2025.112985 (PMC12270803; doi:10.1016/j.isci.2025.112985)
Supplement: Document S1. Tables S1 and S2 [file mmc1.pdf]

iScience, Volume *28*

Supplemental information

Progressive gut microbiota shifts and functional alterations across aging stages and frailty in mice

Xinwei Jiao, Hongyu Li, Ting Wang, Hongchen Fu, Shiwu Wang, Hong Liu, Lei Wang, Xiuyun Li, Aijun Deng, and Zhijie Li

Supplementary Table S1. Concentrations of Eight SCFAs in Fecal Samples from Young-Adult and Frailty Mice								
**Sample ID**	**Acetic acid**	**Propanoic acid**	**Isobutyric acid**	**Butanoic acid**	**Isovaleric acid**	**Valeric acid**	**Isohexanoic acid**	**Hexanoic acid**
Young-adult 1	110.8669	58.4248	6.163	63.6222	4.9707	12.9793	0.2016	0.6208
Young-adult 2	106.3057	80.2088	5.6926	71.131	4.3606	6.3797	0.1592	0.3904
Young-adult 3	84.8333	88.4187	7.0422	80.1275	5.0626	7.2704	0.1551	0.3539
Young-adult 4	83.5132	53.172	6.3194	90.9484	6.0097	16.5587	0.176	0.7425
Young-adult 5	66.5369	54.0955	4.2783	63.1562	3.3006	9.1317	0.1822	0.3853
Young-adult 6	65.4735	58.632	4.3849	59.7285	2.82	6.2778	0.1755	0.4747
Young-adult 7	65.1064	55.0981	4.2783	45.4717	3.3366	7.1931	0.18	0.3585
Young-adult 8	64.3325	44.634	3.2017	49.9472	2.3164	6.7493	0.1336	0.3883
Frailty 1	76.1966	56.8353	5.8646	54.4342	5.2122	9.3157	0.1367	0.4127
Frailty 2	69.8859	41.9853	5.221	43.787	4.9277	12.2639	0.1841	0.5201
Frailty 3	67.1862	51.4666	5.9275	48.8126	6.19	12.1473	0.2603	0.6745
Frailty 4	56.8299	53.9544	4.4561	34.0792	3.2068	4.5127	0.1262	0.3598
Frailty 5	51.8088	46.4785	5.6219	42.1098	4.4937	9.2141	0.1423	0.4197
Frailty 6	51.5673	40.0086	5.2478	44.9669	4.9652	7.8398	0.1281	0.4439
Frailty 7	49.2262	40.6774	4.2959	57.1756	4.0572	7.468	0.1375	0.4564
Frailty 8	49.1707	44.6798	4.2754	54.7731	3.2508	8.3134	0.1507	0.4586

Supplementary Table S2. Sample Raw and Filtered Read Counts for Each Aging Stage

Sample ID	Raw Read Count	Filtered Read Count
Young-adult 1	46222	37303
Young-adult 2	45573	32924
Young-adult 3	42744	34581
Young-adult 4	54611	43698
Young-adult 5	47253	38786
Young-adult 6	47459	38478
Young-adult 7	48155	37313
Young-adult 8	33249	26570
Young-adult 9	45847	33084
Young-adult 10	38922	31217
Middle-aged 1	40342	30687
Middle-aged 2	74852	54649
Middle-aged 3	56893	42844
Middle-aged 4	36840	29078
Middle-aged 5	49849	37581
Middle-aged 6	43031	31270
Middle-aged 7	41774	32226
Middle-aged 8	38244	29341
Middle-aged 9	43012	36081
Middle-aged 10	46607	34113
Senescent 1	52439	44900
Senescent 2	63216	56861
Senescent 3	61627	51311
Senescent 4	50520	45726
Senescent 5	51601	42311
Senescent 6	47161	40023
Senescent 7	41871	37810
Senescent 8	53933	44166
Senescent 9	53109	43208
Senescent 10	51188	44080
Frailty 1	57107	45675
Frailty 2	53732	44857
Frailty 3	47972	39992
Frailty 4	58584	50598
Frailty 5	62304	51814
Frailty 6	58951	48535
Frailty 7	61238	51974
Frailty 8	48947	43873
Frailty 9	48255	45250
Frailty 10	54028	49862
